# Effects of Dietary Protease at Different Levels of Probiotics on the Growth Potential, Enzyme Activities, Physiological and Hematoimmune Responses of Striped Catfish (*Pangasianodon hypophthalmus* (Sauvage, 1878))

**DOI:** 10.3390/ani16213431

**Published:** 2026-10-30

**Authors:** Ayesha Tanveer, Noor Khan, Mahroze Fatima, Hafiz Abdullah Shakir, Wazir Ali, Ayesha Khizar, Samuel A. M. Martin, Simon J. Davies

**Affiliations:** 1 Institute of Zoology, University of the Punjab, Lahore 54590, Pakistan; 2 Scottish Fish Immunology Research Centre, School of Biological Sciences, University of Aberdeen, Aberdeen AB24 3FX, UK; 3 Department of Fisheries and Aquaculture, University of Veterinary and Animal Sciences, Lahore 54000, Pakistan; 4 Aquaculture and Nutrition Research Unit, University of Galway, H91 TK33 Galway, Ireland

**Keywords:** striped catfish, probiotics, protease, growth, digestive and antioxidant enzymes, Immune indices

## Abstract

Probiotics and exogenous enzymes play essential roles in the growth and health of aquatic animals. This study explored the interactive effects of probiotics (Prob) and exogenous protease (Prot) on the growth indices, digestive and antioxidant enzyme activities, body composition, hematoimmune parameters and serum biochemical indices of striped catfish (*n* = 600, ≈10.17 ± 0.03 g). In this context, ten diets were formulated with a crude protein level of 35.23% by incorporating Prob (0.0, 1.0, 2.0, 3.0, and 4.0 g/kg) at each Prot level (0.5 and 1.0 g/kg). After a 14-day acclimatization period, the fish were stocked in ten groups, each in triplicate (20 fish/aquaria (40 × 40 × 70 cm, i.e., width × depth × length)) in a 2 × 5 factorial design and fed for 90 days at 3% of their body weight. The results of the current study revealed significant improvements in growth performance with Prob supplementation at both Prot levels (0.5 and 1.0 g/kg). At 0.5 g/kg Prot, FBW and AWG increased with increasing Prob supplementation, reaching their highest mean values at 4.0 g/kg Prob. However, WG% was highest (498.49%), and FCR was lowest (1.33) at 2.0 g/kg Prob, with no substantial further improvement at higher Prob levels. In contrast, at 1.0 g/kg Prot, the optimal Prob level shifted to 1.0 g/kg, resulting in a WG% of 486.99% and an FCR of 1.37. Similarly, the main effect and interactive effect were significant for digestive and antioxidant enzymes in response to dietary Prot and Prob. However, the levels of malondialdehyde (MDA), white blood cells (WBCs), and liver enzymes significantly decreased, reversing the growth trend. Furthermore, hematoimmunological parameters, including red blood cell (RBC) count, hemoglobin (Hb) concentration, hematocrit (HCT), lysozyme activity, and immunoglobulin M (IgM), showed the highest mean values at 4.0 g/kg Prob with 0.5 g/kg Prot, although the differences among the 2.0, 3.0, and 4.0 g/kg Prob treatments were not statistically significant. At 1.0 g/kg Prot, these parameters generally showed the highest mean values at 1.0–2.0 g/kg Prob. Moreover, the proximate analysis (crude protein, crude fat, moisture and ash) was not significantly (*p* > 0.05) affected by either supplement. The polynomial broken-line regression analysis based on WG% indicated that the optimum Prob level with 0.5 g/kg protease was 1.46 g/kg, whereas at 1.0 g/kg protease supplementation, the optimal probiotic level was estimated at 1.39 g/kg. In conclusion, dietary supplementation with Prob and Prot enhances the growth and health status of *P*. *hypophthalmus*, suggesting a sustainable and economical approach for intensive catfish farming.

## Simple Summary

Aquaculture is an important source of nutritious food, but improving fish growth and health while keeping feed costs low remains a major challenge. This study investigated the combined effects of probiotics, beneficial microorganisms, and protease, a digestive enzyme, on the growth and health of striped catfish. Fish were fed diets containing different levels of probiotics (0.0, 1.0, 2.0, 3.0, and 4.0 g/kg) paired with two levels of protease (0.5 and 1.0 g/kg) for 90 days. Growth performance improved with probiotic supplementation at 0.5 g/kg protease, with FBW and AWG reaching their highest mean values at 4.0 g/kg probiotics, while the highest weight gain percentage (498.49%) and lowest feed conversion ratio (1.33) were recorded at 2 g/kg probiotics, with no significant further improvement in WG% and FCR at higher probiotic levels (3.0–4.0 g/kg). At 1.0 g/kg protease, 1.0 g/kg probiotics produced the best growth performance, with a weight gain percentage of 486.99% and an FCR of 1.37. In addition, both additives also improved digestive enzyme activities, antioxidant status, and immune responses of striped catfish. However, they did not significantly change the basic nutritional composition of the fish. In conclusion, combining probiotics and protease in fish diets may provide a practical, economical, and sustainable approach for improving the production and health of striped catfish in intensive aquaculture.

## 1. Introduction

The demand for sustainable and cost-effective aquatic food production has increased, which has accelerated the substitution of animal protein with plant protein sources in aquafeeds due to the shortage and high cost of fishmeal [1]. Although plant-based ingredients are economical and readily available, their inclusion at high levels in aquafeeds is often associated with nutritional constraints such as digestibility and nutrient absorption, as well as the presence of antinutritional factors (ANFs) and amino acid imbalances [2,3]. To address these issues, various nutritional strategies have been developed, such as the use of exogenous enzymes, prebiotics, nutraceuticals, symbiotic, plant extracts and probiotics, which have therefore been investigated to improve nutrient utilization and maintain fish health. Among these, probiotics and exogenous enzymes have received considerable attention because of their potential to improve feed utilization, growth performance, and physiological health in aquaculture species [4,5,6].

Exogenous protease enzymes increase the utilization of plant protein sources by degrading large protein molecules into bioavailable amino acids, increasing nutrient bioavailability, and mitigating the effects of ANFs [2,7]. Although proteases are naturally present in the digestive tract of fish, their endogenous activity may become limited under certain circumstances. Therefore, dietary supplementation with exogenous protease can compensate for this deficiency [8]. Various studies have shown that dietary protease supplementation improves feed conversion efficiency (FCR), growth performance and nutrient digestibility in fish [9,10]. Similarly, probiotics have gained considerable attention as promising feed additives in aquaculture because they can regulate gut microbial communities, enhance nutrient utilization, and strengthen immune function [11,12,13,14]. The beneficial effects of probiotics on growth performance and health status have been reported in several fish species, including *Oreochromis niloticus* [15], *Catla catla* [16], and *Acipenser baerii* [17]. These potential benefits highlight the importance of investigating dietary probiotics and protease in aquaculture species.

Among economically important aquaculture species, striped catfish (*Pangasianodon hypophthalmus* (Sauvage, 1878)) is an omnivorous species whose feeding habits change during ontogenetic development. Juveniles exhibit a greater preference for animal-based food items and may show cannibalistic behavior, whereas adults can utilize plant-based feed ingredients more readily [18]. Economically, striped catfish contributed approximately 5.1% to global inland finfish aquaculture production and accounted for about 40% of global catfish production [19]. This species plays an important role in aquaculture because of its rapid growth, efficient feed conversion, adaptability to diverse environmental conditions, market demand, acceptable flesh quality, and suitability for intensive farming [4]. Striped catfish is a recently introduced aquaculture practice in Pakistan and is receiving increasing attention as a promising aquaculture species due to its desirable production traits, high yield potential and consumer acceptance [20,21]. Due to its higher productivity and faster growth rate, striped catfish culture has spread in many countries of South and Far Eastern Asia, including Pakistan. Improving the growth, feed efficiency, and health of striped catfish can therefore enhance farm profitability and strengthen aquaculture’s contribution to food security [22].

Previous studies have reported the synergistic role of probiotics with other exogenous enzymes (phytase, xylanase, amylase, beta-glucanase, lipase and cellulase) in improving growth, feed efficiency, and immunophysiological status in fish species, including *Oreochromis niloticus*, *Acipenser baerii*, and *Oncorhynchus mykiss* [23,24,25]. Despite growing interest in dietary probiotics and exogenous enzymes, their interactive effects, particularly in diets containing plant-based ingredients, remain insufficiently studied in striped catfish. Therefore, the supplementation of probiotics and exogenous enzymes in aquafeeds should be considered an effective strategy to improve nutrient utilization and support the sustainable production of striped catfish. Although the individual benefits of probiotics and proteases have been widely studied, findings regarding their interactive effects on plant-based diets for this species are not available. Accordingly, this study aimed to evaluate the interactive effects of dietary probiotics and exogenous protease on growth performance, digestive and antioxidant enzyme activities, body composition, hematoimmune parameters, and serum biochemical indices of striped catfish.

## 2. Materials and Methods

### 2.1. Ethical Approval Statement

Ethical approval for the study was obtained from the Departmental Ethics Review Committee (DERC) of the Institute of Zoology, University of the Punjab, Lahore, Pakistan (Letter No. 171), protocol code: D/171/FIMS, date: 11 December 2025.

### 2.2. Preparation of Experimental Diets

The experiment followed a 2 × 5 factorial design comprising ten experimental diets with a 35.23% crude protein level, containing two dietary protease inclusion levels (0.5 and 1.0 g kg^−1^; CIBENZA DP^®^, 600,000 U g^−1^) and five probiotics inclusion levels (0.0, 1.0, 2.0, 3.0, and 4.0 g kg^−1^) of Compro Napro™ [PR-24] Aqua Probiotics (China), containing *Bacillus licheniformis* (5.0 × 10^9^ CFU g^−1^), *Bacillus subtilis* (5.0 × 10^9^ CFU g^−1^), and *Enterococcus faecalis* (5.0 × 10^8^ CFU g^−1^). The probiotic-free treatments at each protease level served as reference diets for evaluating the effects of probiotic supplementation under the respective protease conditions. Protease was included at both levels as part of the formulation strategy for the plant-ingredient-based diets. All feed ingredients (Table 1), including probiotics and protease, were milled, accurately weighed according to the formulation and thoroughly mixed. An appropriate quantity of water (≈15%) was added to obtain a homogeneous dough, and the dough was blended with predetermined quantities of fish oil, vitamin premix, and mineral premix to ensure a uniform distribution of nutrients (Table 1). The resulting mixture was pelletized via a laboratory pelletizer machine (Anex, AG- 3060) to produce uniformly sized pellets. The pellets were air-dried at room temperature until a constant moisture level was achieved, labeled according to their respective treatments, packed in airtight polyethylene containers, and stored at 4 °C until use.

### 2.3. Water Quality Monitoring, Procurement, Site and Acclimation of Experimental Species

Striped catfish were procured from a private fish hatchery and transported to the Fish Research Farm, PU, Lahore. Before the beginning of the feeding trial, the fish were acclimatized to laboratory conditions for 15 days in a concrete tank (6 ft diameter × 3 ft depth). To minimize the risk of ectoparasitic infections, the fish were subjected to a prophylactic dip of potassium permanganate (5 mg L^−1^) for 15 min before stocking. Following the acclimatization period, striped catfish fingerlings were selected via a simple random sampling method, individually weighed, and stored in the experimental units. The feeding trial was conducted for 90 days in 30 glass aquaria (70 × 40 × 40 cm; length × width × depth), each containing 100 L of dechlorinated borewell water. A total of 600 fingerlings with an initial body weight of 10.17 ± 0.03 g were randomly stocked at 20 fish per aquarium, with three replicate aquaria assigned to each of the 10 dietary treatments, resulting in 60 fish per treatment. Throughout the experiment, continuous aeration was supplied to maintain adequate dissolved oxygen levels, and a constant water volume was maintained in each tank. The experimental diets were offered to the fish by hand twice daily at 09:00 and 17:00 h, at approximately 3% of their body weight. The feeding rates were adjusted biweekly on the basis of the fish weight measurements. During the experimental period, one-third of the water in each aquarium was replaced daily with fresh aerated water. Uneaten feed was siphoned out after one hour of feeding, dried, and weighed to determine feed intake. The physicochemical parameters were maintained within the normal range throughout the 90-day experimental period. The water temperature was measured via a digital thermometer (TP-300) and ranged from 24 to 28 °C. The dissolved oxygen content was determined via a dissolved oxygen meter (DO-9100) and remained between 5 and 6 mg L^−1^. The water pH was measured via a pH meter (AS-218, China) and ranged from 7.31 to 7.74. The ammonia concentration was determined via an ammonia test kit (HI-3824) and remained at 0.14 ± 0.02 mg/L throughout the study.

### 2.4. Fish Harvesting

At the end of the 90-day feeding trial, all fish were anesthetized with MS-222 and harvested [26]. Samples from fish within each replicate aquarium were pooled and analyzed separately for each treatment. The data from the three replicate aquaria per treatment (*n* = 3) were used for statistical analysis.

For this purpose, five fish from each replicate were randomly selected for whole-body proximate composition analysis. Seven fish from each replicate were used for enzyme analyses; intestine samples were collected for digestive enzyme assays and liver samples collected for antioxidant enzyme assays. For hematological analysis, blood was collected from the caudal vein of four fish per replicate using 3 mL sterile syringes (Amson Vaccines Pharma, Lahore, Pakistan) and transferred into EDTA (ethylenediaminetetraacetic acid) vials (AT tubes). For serum biochemical analysis, blood samples from an additional four fish per replicate were collected into serum collection tubes, allowed to clot at room temperature for 30 min, and centrifuged at 5000 rpm for 15 min. The separated serum was carefully collected for subsequent analyses. All tissue and serum samples were stored at −20 °C until further analysis.

### 2.5. Growth Performance

During the experimental trial duration, the fish from each aquarium were netted and then weighed at two-week intervals to monitor growth performance and adjust the feeding ratio. The following growth indices were calculated via standard formulas:

Weight gain = final weight − initial weight

$$\text{Weight Gain}\;\%=\frac{\text{Final Weight}-\text{Initial Weight}}{\text{Initial Weight}}\times 100$$

$$\mathrm{Feed Conversion Ratio}=\frac{\text{Feed given}\left(\text{g dry weight}\right)}{\text{Body weight gain}\left(\text{g wet weight}\right)}$$

$$\text{Specific}\;\text{growth}\;\text{rate}\;(\%\;{\text{day}}^{-1})=\frac{{\mathrm{Log}}_{e}\text{Final}\;\text{weight}\;-\;{\mathrm{Log}}_{e}\text{Initial}\;\text{weight}}{\text{Number}\;\text{of}\;\text{days}}\times 100$$

$$\text{Fish}\;\text{Survival}\;(\%)=\frac{\text{Total}\;\text{number}\;\mathrm{of}\;\text{fish}\;\text{harvested}}{\text{Total}\;\text{number}\;\text{of}\;\text{fish}\;\text{stocked}}\times 100$$

FI (g/fish) = given feed − uneaten feed

### 2.6. Proximate Analysis of Fish and Feed

The proximate compositions of whole-body fish samples and experimental diets were determined following standard analytical procedures of AOAC [27]. The samples of fish and feed were dried at 105 °C for 24 h in a hot air oven (WGL-50ABE). The crude protein content was determined from the nitrogen concentration via a conversion factor of 6.25 (N × 6.25), with nitrogen measured via a micro-Kjeldahl apparatus (AKN-01S, BIOBASE Group, Jinan, China). Moreover, the fat content was analyzed via petroleum ether extraction via a Soxhlet apparatus (LSOA-A12). The ash content of the samples was determined by incineration in a muffle furnace (CY-M1700-2 L) at 600 °C for 6 h until a constant weight was reached.

### 2.7. Determination of Digestive Enzyme (Protease, Amylase, and Lipase) Activities

Digestive enzyme activities were analyzed in intestinal tissue homogenates following the methods described by Khizar and Bano [28,29]. Briefly, fish intestine samples were collected, washed with distilled water, weighed and homogenized in ice-cold sucrose solution (43 g sucrose in 500 mL distilled water) to obtain a 5% (*w*/*v*) tissue homogenate via a Teflon-coated tissue homogenizer (A-88, Nanjing AMIS Medical Technology Co., Ltd., Nanjing, China). All homogenization procedures were carried out under chilled conditions to prevent enzyme denaturation. The homogenate was subsequently centrifuged at 15,000× *g* for 15 min at 4 °C (SCILOGEX^DM0408^). The enzyme supernatant fraction was subsequently collected and used for subsequent enzyme assays. Amylase activity was determined using the dinitrosalicylic acid (DNS) method. Briefly, 0.5 mL of enzyme extract was incubated with 0.5 mL of starch solution at 37 °C for 30 min, followed by the addition of 1 mL DNS reagent and heating for 5 min. Absorbance was measured at 540 nm after fivefold dilution, and activity was calculated using a maltose standard curve. Protease activity was determined using the casein digestion method. Enzyme extract (0.5 mL) was incubated with 0.5 mL casein solution at 37 °C for 20 min. The reaction was stopped by adding 1 mL trichloroacetic acid (TCA), followed by incubation at 2 °C for 1 h and centrifugation. The supernatant was filtered, and absorbance was measured at 280 nm and calculated using a tyrosine standard curve. Lipase activity was measured using the p-nitrophenyl palmitate (pNPP) assay. The reaction mixture containing 0.9 mL pNPP solution, 0.1 mL enzyme extract, and 0.1 mL phosphate buffer was incubated at 37 °C for 30 min. Absorbance was measured at 410 nm, and lipase activity was expressed as μmol p-nitrophenol released min^−1^ mg protein^−1^.

### 2.8. Determination of Antioxidant Enzyme Activities

Liver samples were homogenized in ice-cold phosphate buffer (pH 6.5; 1 g tissue in 4 mL buffer) and centrifuged at 9500× *g* for 20 min at 4 °C. The supernatant was stored at −80 °C until analysis. Catalase (CAT) activity was determined by monitoring the decomposition of hydrogen peroxide (H_2_O_2_) at 240 nm following Chance and Maehly [30]. The reaction contained 1.95 mL phosphate-buffered H_2_O_2_ substrate and 50 µL enzyme extract. Absorbance was recorded at 1 min intervals over 3 min, and CAT activity was calculated from the change in absorbance per minute. Glutathione peroxidase (GPx) activity was assessed spectrophotometrically at 470 nm using a guaiacol–H_2_O_2_ substrate solution following the method of Civello [31]. The reaction contained 3 mL substrate solution and 60 µL enzyme extract, with absorbance recorded after 3 min. Enzyme activity was calculated from the change in absorbance per minute. SOD activity was determined by measuring the inhibition of NBT photoreduction following the method of Giannopolitis and Ries [32]. The reaction mixture was illuminated using a 30 W light source, and absorbance was measured at 560 nm. SOD activity was expressed as percentage inhibition relative to the blank. Malondialdehyde (MDA) content was determined using the TBARS method following the method of Gatta [33]. Liver homogenate was incubated with ascorbic acid at 37 °C for 25 min, followed by HCl and thiobarbituric acid (TBA) treatment at 95 °C for 30 min. After adding trichloroacetic acid (TCA) and centrifugation at 3000 rpm for 5 min, absorbance was measured at 530 nm. MDA content was calculated using a standard curve and expressed as µg MDA equivalents/mg sample.

### 2.9. Blood and Serum Biochemical Parameters

Hematological parameters, including red blood cell count (RBC), white blood cell count (WBC), hemoglobin concentration (Hb), and hematocrit (HCT), were determined from fish blood samples collected in EDTA-containing vials using an automated hematology analyzer (XN-330™, Sysmex Corporation, Kobe, Japan). Serum biochemical parameters were analyzed by commercial assay kits according to the manufacturer’s instructions, with the following catalog numbers: ALP (MBS-2567975), ALT (MET-5123), AST (MET-5127), etc.

### 2.10. Determination of Immunological Responses

For the assessment of immune parameters, serum samples were analyzed for lysozyme (Lyz) activity and total immunoglobulin M (IgM) levels. The activity of Lyz was calculated by measuring the lysis of a *Staphylococcus aureus* suspension (optical density: 0.6–0.7 at 450 nm) in a buffer solution (phosphate buffer) after the mixture was incubated with 100 µL of serum at 40 °C, following the methods of Ellis [34], while total immunoglobulin (IgM) concentration was determined according to Anderson and Siwicki using polyethylene glycol (PEG) precipitation. Briefly, serum samples were treated with PEG to precipitate immunoglobulins and incubated at room temperature. The samples were then centrifuged, and the protein concentration of the resulting supernatant was determined spectrophotometrically at 595 nm. IgM concentration was calculated from the difference between the total protein concentration of the untreated serum and the protein concentration of the PEG-treated supernatant [35].

### 2.11. Statistical Analysis

Statistical analyses were performed via SPSS software (version 27.0). Data normality and homogeneity of variance were assessed via the Shapiro–Wilk test and Levene’s test, respectively. All the data are presented as the means ± standard deviations (SDs) of three replicates (*n* = 3). The effects of dietary probiotic level, protease levels, and their interactive effects were evaluated via two-way analysis of variance (ANOVA; IBM SPSS Statistics 27.0 When a significant probiotic × protease interaction was detected, the effects were analyzed via one-way ANOVA to compare the probiotic levels within each protease level, followed by Tukey’s honestly significant difference (HSD) test for multiple comparisons. Broken-line regression analysis was performed to estimate the optimum dietary probiotic level at each protease supplementation level. All the differences in terms of means and standard deviations were considered statistically significant at *p* < 0.05.

## 3. Results

### 3.1. Growth Indices of Striped Catfish

Growth performance was significantly influenced by dietary Prob and Prot supplementation, with significant Prob × Prot interactions for final body weight (FBW), average weight gain (AWG), weight gain percentage (WG%), and specific growth rate (SGR) (all *p* < 0.001; Table 2). Compared with the reference diets (0.0 g/kg Prob) at each Prot level, probiotic supplementation improved growth, although the response depended on the dietary Prot concentration. At 0.5 g/kg Prot, FBW and AWG increased with increasing Prob supplementation, reaching their highest mean values at 4.0 g/kg Prob. However, WG% was highest at 2.0 g/kg Prob, with no significant differences among the 2.0, 3.0, and 4.0 g/kg treatments. SGR also increased up to 2.0 g/kg Prob, without significant further improvement at higher inclusion levels. Similarly, FCR decreased up to 2.0 g/kg Prob, with no significant differences between the 2.0, 3.0, and 4.0 g/kg treatments.

**Figure 1:**
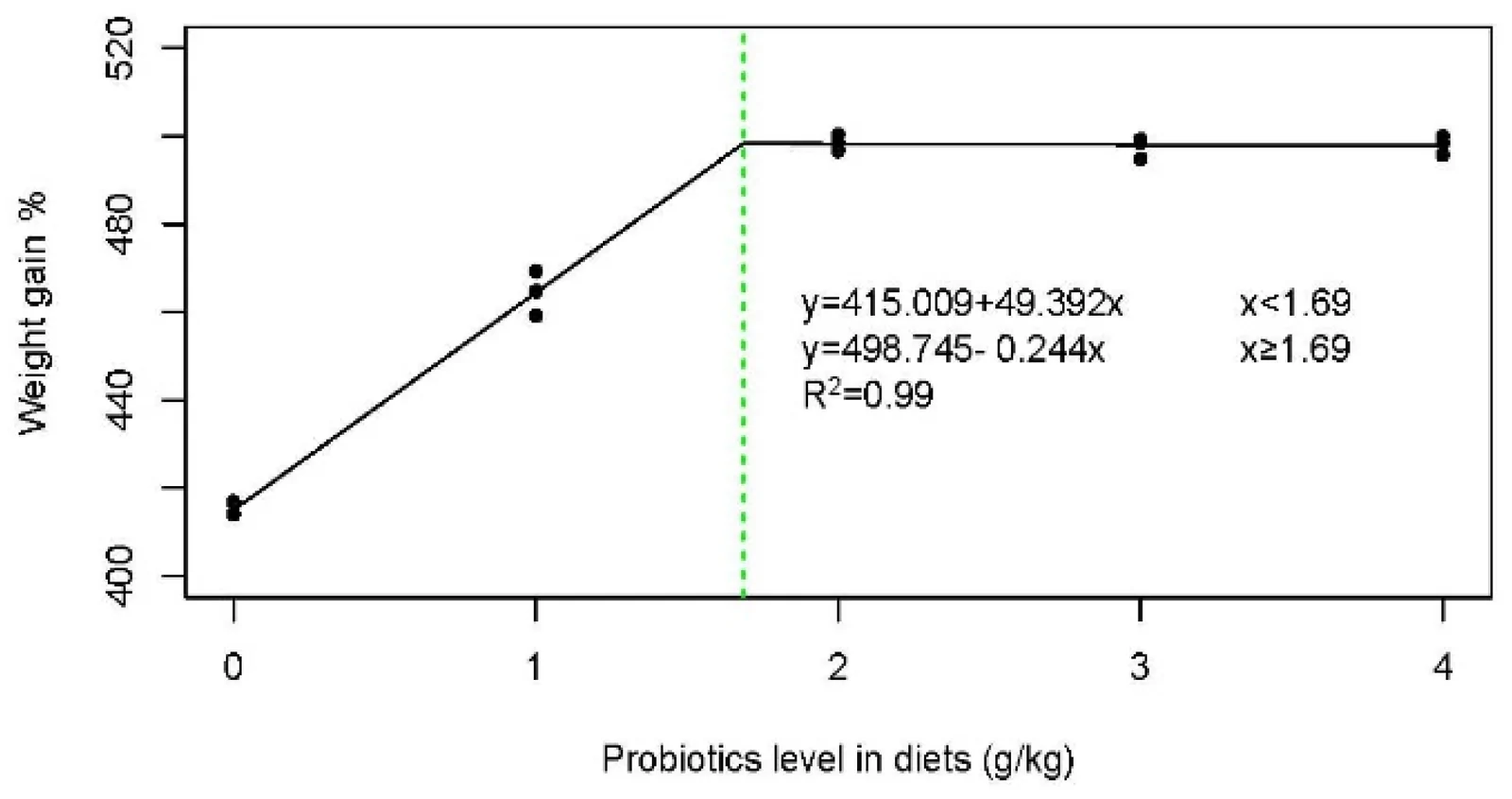
Optimum probiotic concentration of 0.5 g/kg Protease.

**Figure 2:**
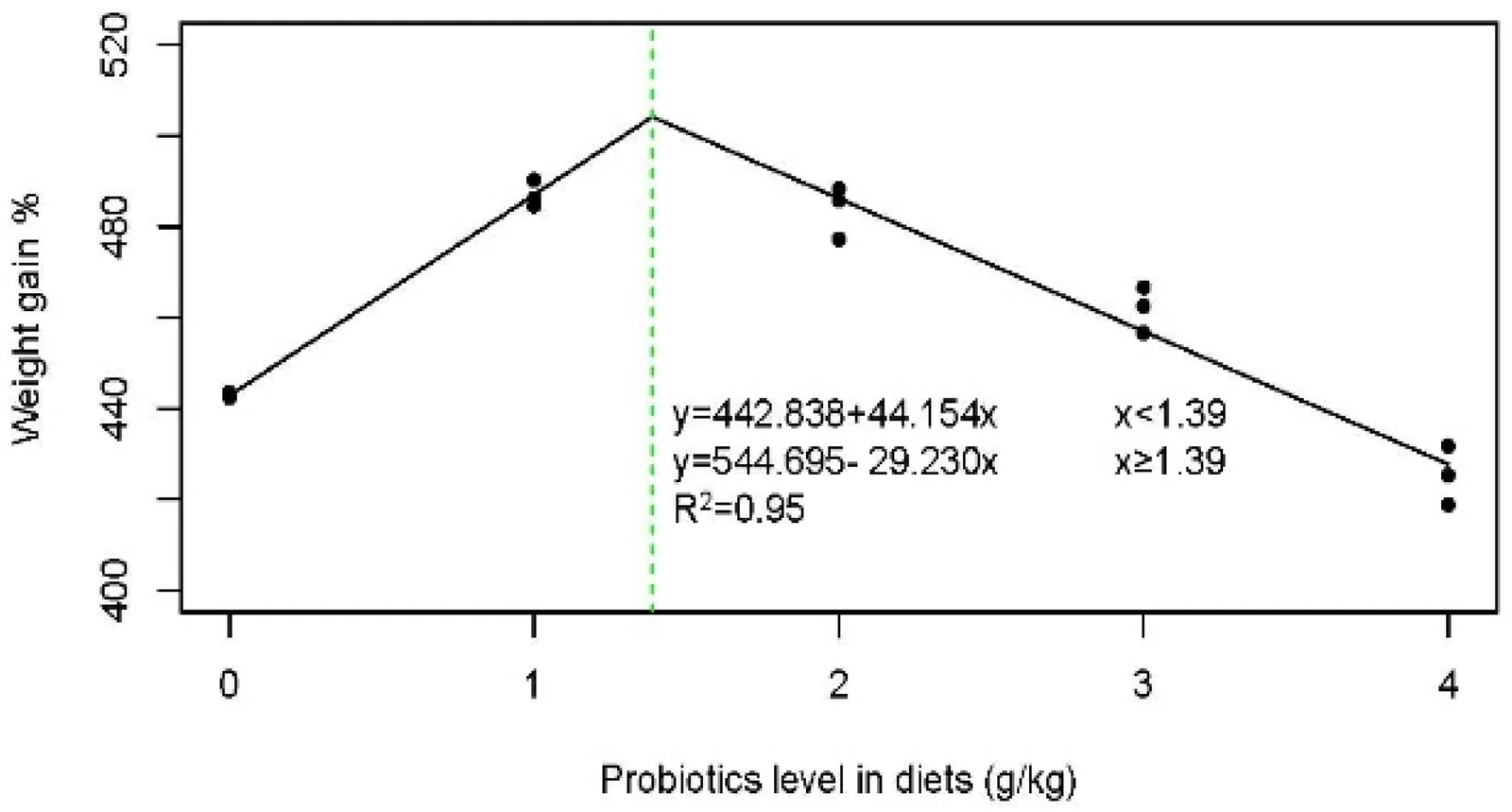
Optimum probiotic concentration of 1.0 g/kg Protease.

At 1.0 g/kg Prot, the highest mean values for FBW, AWG, WG%, and SGR were observed at 1.0 g/kg Prob, although these did not differ significantly from those at 2.0 g/kg Prob. Nevertheless, these growth indices declined at higher inclusion levels (3.0 and 4.0 g/kg Prob). Conversely, FCR was lowest at 1.0–2.0 g/kg Prob and increased at higher inclusion levels. Moreover, broken-line regression analysis based on WG% estimated the optimal Prob inclusion levels at 1.69 g/kg for the diet containing 0.5 g/kg Prot (Figure 1) and 1.39 g/kg for the diet containing 1.0 g/kg Prot (Figure 2). Furthermore, survival rate (SR) was not significantly affected by the individual or interactive effects of Prob and Prot (*p* > 0.05).

### 3.2. Proximate Analysis

Dietary Prob and Prot supplementation, either individually or in combination, had no significant effects on the proximate composition of striped catfish (*p* > 0.05; Table 3). Moisture content ranged from 74.97% to 75.82%, and crude protein content ranged from 16.89% to 17.75% across treatments. Similarly, crude fat and ash contents varied from 3.66% to 4.11% and from 2.29% to 3.23%, respectively. No significant differences were observed among dietary treatments for any of the measured proximate composition parameters.

### 3.3. Intestinal Digestive Enzymes

Intestinal amylase, protease, and lipase activities were significantly affected by dietary Prob and Prot supplementation and their interaction (Table 4; *p* < 0.05 for all effects) in striped catfish. Compared with the reference diet (0.0 g/kg Prob) at 0.5 g/kg Prot, the mean activities of all three enzymes increased with increasing Prob supplementation, reaching their highest values at 4.0 g/kg Prob. However, no significant differences were observed among the 2.0, 3.0, and 4.0 g/kg Prob treatments for any of these enzymes. At 1.0 g/kg Prot, the highest mean amylase (1.53 U/mg protein), protease (8.56 U/mg protein), and lipase (2.68 U/mg protein) activities were observed at 1.0 g/kg Prob. Activities at 2.0 g/kg Prob remained statistically comparable to those at 1.0 g/kg, whereas higher Prob levels resulted in lower enzyme activities in striped catfish.

### 3.4. Antioxidant Enzyme Activities

Dietary Prob and Prot supplementation and their interaction significantly (*p* < 0.05) affected SOD, CAT, GPx, and MDA levels (Table 5). At 0.5 g/kg Prot, SOD, CAT, and GPx activities increased with increasing Prob supplementation, with no significant differences among the 2.0, 3.0, and 4.0 g/kg Prob treatments. The highest mean SOD (5.05 U/mg protein), CAT (79.42 U/mg protein), and GPx (93.55 μU/mg protein) activities were recorded at 4 g/kg Prob. In contrast, at 1.0 g/kg Prot, the highest mean activities of SOD (5.23 U/mg protein), CAT (79.49 U/mg protein), and GPx (94.34 μU/mg protein) were observed at 1.0 g/kg Prob, with activities at 2.0 g/kg Prob not differing significantly. Activities subsequently declined at higher Prob levels. MDA levels showed an opposite response. At 0.5 g/kg Prot, MDA decreased from 3.56 to 2.53 mg/g protein as Prob supplementation increased from 0.0 to 4.0 g/kg, with no significant differences among the 2.0, 3.0, and 4.0 g/kg treatments. At 1.0 g/kg Prot, MDA was lowest at 1.0–2.0 g/kg Prob (2.42–2.45 mg/g protein) and increased at 3.0–4.0 g/kg Prob.

### 3.5. Blood Profile of Striped Catfish

The hematological responses differed according to dietary Prob inclusion and Prot level (Table 6). At 0.5 g/kg Prot, supplementation with 2.0 g/kg Prob resulted in RBC, HB, and HCT having the highest mean values of 2.51 × 10^6^ μL^−1^, 8.52 g/dL, and 22.40%, respectively. Although the highest mean values were recorded at 4.0 g/kg Prob, the differences among 2.0, 3.0, and 4.0 g/kg Prob were not statistically significant for the respective parameters. Moreover, at 1.0 g/kg Prot, the highest mean RBC count (2.55 × 10^6^ μL^−1^) and HB concentration (8.57 g/dL) were observed at 1.0 g/kg Prob, while HCT reached its highest mean value (22.47%) at 2 g/kg Prob. Furthermore, WBC counts showed a different trend: at 0.5 g/kg Prot, the lowest mean count (5.42 × 10^3^ μL^−1^) was recorded at 2.0 g/kg Prob, whereas at 1.0 g/kg Prot, the lowest mean (5.34 × 10^3^ μL^−1^) occurred at 1.0 g/kg Prob. The main effect of Prot on WBC count was not significant (*p* = 0.902), while Prob and its interaction with Prot were significant (*p* < 0.001).

### 3.6. Serum Immunological and Biochemical Parameters

Serum biochemical and immunological responses varied with dietary Prob inclusion and Prot concentration (Table 7). At 0.5 g/kg Prot, ALP, ALT, and AST activities decreased with increasing Prob supplementation up to 2.0 g/kg, and increased thereafter with no significant differences among the 2.0, 3.0, and 4.0 g/kg treatments. In contrast, at 1.0 g/kg Prot, the lowest mean ALP (28.27 U/L), ALT (79.05 U/L), and AST (14.54 U/L) activities were recorded at 1.0 g/kg Prob, with ALT and AST values at 2.0 g/kg Prob not differing significantly from those at 1.0 g/kg. However, these enzyme activities increased at higher Prob inclusion levels. The main effects of Prob and the Prob × Prot interaction were significant for all three biochemical parameters (*p* < 0.001), whereas the individual effect of Prot was not significant (*p* > 0.05). Regarding immunological responses, Lyz activity and IgM concentration significantly increased with Prob supplementation at both Prot levels, with significant main effects of Prob, Prot, as well as their interaction (Prob × Prot; *p* < 0.001). At 0.5 g/kg Prot, the highest mean Lyz activity (65.19 ± 0.03 U/mL) was recorded at 4.0 g/kg Prob; however, it did not differ significantly from the values at 2.0 and 3.0 g/kg Prob. Similarly, serum IgM concentration increased from 3.18 mg/mL (reference diet) to 5.81 mg/mL at 2.0 g/kg Prob, and then decreased with an increase in Prob levels.

## 4. Discussion

Plant-derived proteins are increasingly used as alternatives to animal proteins because of their lower cost, availability and sustainability advantages. However, omnivores as well as carnivorous fish often utilize plant proteins inefficiently because of their limited digestive capacity and sensitivity to plant-derived antinutritional factors [36]. Dietary supplementation with exogenous proteases may increase plant protein hydrolysis and nutrient availability, whereas probiotics can improve digestion, gut health, and immune function. Therefore, the use of proteases and probiotics represents a promising strategy to improve plant protein utilization in carnivorous fish. The results of the present investigation revealed that the interactive effect of dietary Prob and Prot significantly improved the growth of striped catfish in terms of FW, AWG, WG%, SGR and FCR. Several studies have shown that probiotics can significantly (<0.001) improve the growth performance of aquaculture species [37,38]. They can often act as a combined source of nutrients, vitamins, and digestive enzymes, thereby increasing feed utilization and nutrient absorption. By colonizing the gastrointestinal tract, probiotics establish a balanced gut microbiota, suppress pathogenic microorganisms, and improve digestion and nutrient uptake and assimilation while improving digestibility, appetite, survival, and growth [4,11,12]. Furthermore, dietary exogenous proteases also increase fish growth by increasing feed utilization, digestion and nutrient absorption. They break down complex compounds, increasing nutrient availability, metabolic efficiency and degradation of antinutritional factors [17,39,40]. Therefore, their interactive effects may contribute to improved growth performance. At 0.5 g/kg Prot, the highest mean values for some growth parameters were recorded at 4 g/kg Prob; however, the differences among the 2.0, 3.0, and 4.0 g/kg Prob treatments were not statistically significant. Furthermore, broken-line regression estimated an optimal Prob inclusion level of 1.69 g/kg at this protease level. Interestingly, at 1.0 g/kg Prob, significantly higher growth performance was observed with 1.0 g/kg Prot, indicating that at higher protease levels, lower levels of probiotics are needed. This reduction at higher inclusion levels might be attributed to an imbalance in the gut microbial community due to increased energy demands associated with excessive probiotic supplementation [41]. Moreover, previous studies revealed that supplementation with protease at relatively high levels leads to intestinal damage and metabolic disturbances in other nutrients in the feed [42,43]. Thus, from the previous literature, it was also observed that the optimum supplemental level of both additives (Prob and Prot) has been examined in different fish species, such as *Oreochromis niloticus* [44], *Labeo rohita* [9], *Acipenser baerii* [17], *Clarias batrachus* [26] and *Catla catla* [16]. Notably, this interaction is attributed to dietary supplementation (Prob and Prot), which provides a suitable substrate for the microbiota of the intestine and stimulates appetite, the production of useful metabolites, and the proliferation of beneficial gut bacteria, leading to better growth and mitigating adverse effects on overall health [9,45]. Despite this, the exact mechanisms underlying the interactive effect are not yet known. However, some scientific studies have highlighted the synergistic effects of probiotics with other enzymes in *Oncorhynchus mykiss* [25], *Oreochromis niloticus* [45] and *Channa argus* [46].

Digestive enzyme activity is a key indicator of nutrient utilization efficiency, as it directly reflects the capacity of the gastrointestinal tract to break down and absorb dietary nutrients [47]. In the present study, the digestive enzyme activity significantly increased in response to the interaction effect of Prob and Prot in *P. hypophthalmus.* Probiotics promote a favorable intestinal environment and improve digestion through the production of digestive enzymes. These enzymes facilitate the breakdown of complex nutrients and stimulate endogenous enzyme secretion, thereby increasing overall digestive efficiency in the gut [24,26,44]. Furthermore, the observed increase in digestive enzyme activity may also be attributed to the stimulation of endogenous enzyme production. The secretion of endogenous digestive enzymes is influenced by several factors, including the health status, feeding regime, age, genetic makeup, and environmental conditions of the fish [9]. Consequently, the synergistic supplementation of Prob and Prot resulted in enhanced nutrient digestion and absorption in fish, which is associated with an improved microbial balance in the gastrointestinal tract [46]. The findings of the present study are consistent with those of previous reports on several fish species, such as *Acipenser baerii* [17,23] and *Oreochromis niloticus* [45].

Oxidative stress is a significant health challenge in intensive aquaculture [48]. The findings of the present study revealed that the levels of antioxidant enzymes (CAT, GPx, and SOD) in *P. hypophthalmus* were significantly increased by the interactive effects of Prob and Prot supplementation, with decreased MDA concentrations. Recent studies have indicated that probiotics produce antioxidant metabolites (glutathione, exopolysaccharides, butyrate, and folate) that enhance antioxidant defense mechanisms, improve immunity, and protect against tissue damage caused by oxidative stress [26,49]. Moreover, the reduction in MDA levels (a biomarker of lipid peroxidation) indicates that improved protein digestibility due to protease supplementation may protect fish from intestinal damage and alleviate oxidative stress by reducing free radical-induced damage [8]. Similarly, in previous studies, when probiotics were supplemented with other exogenous enzymes (phytase, protease and carbohydrase) in *O. niloticus,* positive effects were reported [24,50].

Blood parameters serve as reliable indicators of the overall physiological and health status of fish [51]. In the current study, the interaction effect of Prob and Prot supplementation significantly (*p* < 0.05) increased RBC, Hb and HCT levels and reduced WBCs in *P. hypophthalmus*. The elevated level of Hb may be associated with increased oxygen demand resulting from increased oxygen saturation and dissociation rates [52], whereas the increased RBC count indicates an improved physiological and health status of the fish associated with improved antioxidant status and therefore reduced oxidative stress by preventing RBC lysis [26]. Moreover, elevated WBC counts may indicate infection, whereas reduced WBC counts suggest enhanced immunity [17]. Consistent findings have been reported for individual supplementation with Prob and Prot in fish diets [17,45,53,54,55,56].

Serum biochemical parameters are important tools for evaluating liver function and health status [57]. In the present study, the interaction effect of dietary Prob and Prot significantly (*p* < 0.05) decreased liver enzyme activities in *P. hypophthalmus*. The hepatoprotective effects of probiotics may be attributed to their ability to stabilize cellular membranes, reduce tissue damage, enhance anabolic processes, promote protein synthesis and release into the bloodstream [15,58]. It was also observed in our study that the individual effects of dietary Prot supplementation had no significant effect on serum biochemistry. These findings are in accordance with those of studies reporting no significant changes in ALP, AST, or ALT activity in fish [9,10,59]. Our findings revealed that protease had no negative effect on hepatic health or protein metabolism in the experimental fish. However, the interactive effect of Prob and Prot significantly improved liver function, which may be attributed to the hepatoprotective role of probiotics [4,12]. However, research on the interactive effects of dietary Prob and exogenous enzymes on serum liver enzymes in fish is scarce.

The immune response of fish plays a crucial role in health, in which lysozyme (a bacteriolytic enzyme) contributes to bacterial cell wall degradation, whereas IgM (a primary immunoglobulin) plays a central role in the humoral immune response [60]. In the present study, the increased lysozyme activity in probiotic- and protease-supplemented fish may reflect enhanced innate immune defense, as probiotics may enhance lysozyme production and support immune function in fish [61,62,63]. Similarly, the elevated IgM level may be associated with improved protein availability resulting from protease-mediated hydrolysis of complex proteins and antinutritional factors, thereby supporting antibody synthesis [64,65]. The synergistic effect of Prob and Prot observed in this study therefore suggests improved innate and humoral immune readiness, which may contribute to better disease resistance. These findings are consistent with previous reports in sturgeon (Acipenser baerii) showing beneficial effects of probiotics and proteases [17,23].

## 5. Conclusions

The present study revealed that dietary probiotics and exogenous proteases enhance the growth and overall health status of striped catfish. The estimated optimum probiotic supplementation levels were 1.69 g/kg at 0.5 g/kg protease and 1.39 g/kg at 1.0 g/kg protease, indicating that the optimal probiotic inclusion level may vary depending on the dietary protease level. These findings highlight the potential of probiotics and exogenous protease as functional ingredients in aquafeeds for striped catfish. However, this study has several limitations, including the absence of a true control diet and the evaluation of physiological and biochemical responses at a single time point. Future studies should assess these responses at multiple time points throughout the experimental period to better understand the temporal effects of dietary supplementation. Further investigations should explore the underlying mechanisms through which these additives influence gut mucosal barrier function and gut microbiota composition using molecular approaches, including transcriptomic and proteomic analyses of immunity-related pathways. Additionally, future disease-challenge trials should evaluate the effects of these dietary interventions on disease resistance and survival in striped catfish. Future studies should also retain the aquarium as the experimental unit while collecting sufficient individual-level data, where analytically feasible, to quantify both between-aquarium treatment effects and within-aquarium variation, ideally using hierarchical or mixed-effects statistical models.

## Figures and Tables

**Table 1:** Formulation and composition of the experimental diets.

Feed Ingredients (g/kg)										
Ingredients (g/kg)	Prot_0.5_					Prot_1.0_				
	Prob_0.0_	Prob_1.0_	Prob_2.0_	Prob_3.0_	Prob_4.0_	Prob_0.0_	Prob_1.0_	Prob_2.0_	Prob_3.0_	Prob_4.0_
Soybean meal	160	160	160	160	160	160	160	160	160	160
Fish meal	100	100	100	100	100	100	100	100	100	100
Sunflower meal	300	300	300	300	300	300	300	300	300	300
Corn gluten (60%)	100	100	100	100	100	100	100	100	100	100
Canola meal	150	150	150	150	150	150	150	150	150	150
Wheat flour	69.5	68.5	67.5	66.5	65.5	69	68	67	66	65
Fish oil	80	80	80	80	80	80	80	80	80	80
^a^ Vitamins	20	20	20	20	20	20	20	20	20	20
^b^ Minerals	20	20	20	20	20	20	20	20	20	20
^c^ Probiotics	00	01	02	03	04	00	01	02	03	04
^d^ Protease	0.5	0.5	0.5	0.5	0.5	01	01	01	01	01
Total	1000	1000	1000	1000	1000	1000	1000	1000	1000	1000
Nutritional Matrix (%)										
Dry matter	92.11 ± 0.84	91.01 ± 0.55	91.64 ± 0.67	91.70 ± 0.57	92.43 ± 0.01	91.96 ± 0.47	91.01 ± 0.55	91.72 ± 0.55	92.44 ± 0.03	90.80 ± 0.59
Crude protein	34.88 ± 0.44	35.36 ± 0.67	35.20 ± 0.42	35.04 ± 0.80	35.04 ± 0.64	35.74 ± 0.80	35.74 ± 0.11	35.75 ± 0.09	35.79 ± 0.04	35.40 ± 0.44
Crude Fat	7.71 ± 0.66	7.91 ± 0.53	7.88 ± 0.52	7.72 ± 0.19	7.87 ± 0.05	7.80 ± 0.14	7.77 ± 0.15	7.76 ± 0.19	7.76 ± 0.20	7.55 ± 0.24
Ash content	6.65 ± 0.15	6.53 ± 0.21	6.73 ± 0.21	6.71 ± 0.23	6.83 ± 0.12	6.75 ± 0.16	6.84 ± 0.10	6.85 ± 0.11	6.74 ± 0.24	6.60 ± 0.19

^a^ Vitamins premix contains/kg calcium pentothenate (20,000 mg), vitamin B12 (8000 mg), vitamin B6 (5000 mg), vitamin A (16 M.I.U.), vitamin C (16,000 mg), vitamin D3 (4 M.I.U.), folic acid (850 mg), vitamin K3 (6000 mg), nicotinic acid (36,000 mg), vitamin B2 (7000 mg), vitamin B (16,000 mg), and vitamin E (7000 IU), ^b^ minerals mixture contains mg/kg: ZnSO_4_.7H_2_O (121.33), FeSO_4_⋅H_2_O (100.67), cellulose (65), KH_2_PO_4_ (479), CaCO_3_ (316), MgSO_4_.7H_2_O (153), CuSO_4_.5H_2_O (210.67), NaCl (51), MnSO_4_.5H_2_O (116.67) and AlCl_3_.6H_2_O (0.255), ^c^ probiotics: *B. licheniformis* CFU/g (5.0 × 10^9^), *Bacillus subtilis* CFU/g (5.0 × 10^9^) and *Enterococcus faecalis* (5.0 × 10^8^ CFU/g), ^d^ Protease: CIBENZA DP^®^, 600,000 U g^−1^, ^d^ analyzed according to the protocol of the Association of Analytical Chemists (AOAC 2016).

**Table 2:** Interactive effects of dietary protease at different probiotic concentrations on the growth performance of striped catfish.

Parameters	Protease (g/kg)	Probiotics (g/kg) Levels				
		0.0	1.0	2.0	3.0	4.0
IBW (g)	0.5	10.14 ± 0.04	10.15 ± 0.03	10.16 ± 0.04	10.19 ± 0.03	10.19 ± 0.03
	1.0	10.19 ± 0.03	10.17 ± 0.05	10.21 ± 0.06	10.19 ± 0.03	10.16 ± 0.06
FBW (g)	0.5	52.25 ± 0.36 ^Bc^	57.32 ± 0.34 ^Bb^	60.84 ± 0.96 ^Aa^	60.88 ± 0.60 ^Aa^	60.93 ± 0.58 ^Aa^
	1.0	55.33 ± 0.21 ^Ab^	59.73 ± 0.52 ^Aa^	59.60 ± 0.71 ^Ba^	57.26 ± 0.34 ^Bc^	53.38 ± 0.34 ^Bd^
AWG (g)	0.5	42.11 ± 0.32 ^Bc^	47.16 ± 0.37 ^Bb^	50.68 ± 0.91 ^Aa^	50.69 ± 0.57 ^Aa^	50.74 ± 0.55 ^Aa^
	1.0	45.14 ± 0.18 ^Ab^	49.56 ± 0.47 ^Aa^	49.39 ± 0.68 ^Ba^	47.07 ± 0.37 ^Bc^	43.22 ± 0.40 ^Bd^
WG%	0.5	415.00 ± 1.62 ^Bc^	464.40 ± 5.06 ^Bb^	498.49 ± 6.90 ^Aa^	497.48 ± 4.24 ^Aa^	498.00 ± 4.03 ^Aa^
	1.0	442.83 ± 0.61 ^Ab^	486.99 ± 2.90 ^Aa^	483.77 ± 5.79 ^Ba^	461.93 ± 5.01 ^Bc^	425.31 ± 6.44 ^Bd^
SGR (% day^−1^)	0.5	1.82 ± 0.00 ^Bc^	1.92 ± 0.00 ^Bb^	1.98 ± 0.01 ^Aa^	1.98 ± 0.00 ^Aa^	1.98 ± 0.00 ^Aa^
	1.0	1.87 ± 0.00 ^Ab^	1.96 ± 0.00 ^Aa^	1.96 ± 0.01 ^Ba^	1.91 ± 0.00 ^Bc^	1.84 ± 0.01 ^Bd^
FCR	0.5	1.60 ± 0.00 ^Aa^	1.44 ± 0.02 ^Ab^	1.33 ± 0.01 ^Bc^	1.34 ± 0.01 ^Bc^	1.34 ± 0.01 ^Bc^
	1.0	1.51 ± 0.00 ^Bc^	1.37 ± 0.00 ^Bd^	1.38 ± 0.02 ^Ad^	1.45 ± 0.01 ^Ab^	1.57 ± 0.02 ^Aa^
FI (g fish^−1^)	0.5	67.50 ± 0.78	67.93 ± 0.72	68.19 ± 0.52	68.22 ± 0.27	68.22 ± 0.27
	1.0	68.25 ± 0.38	68.20 ± 0.29	68.23 ± 0.34	68.31 ± 0.31	68.22 ± 0.27
SR (%)	0.5	100.00	100.00	100.00	100.00	100.00
	1.0	100.00	100.00	100.00	100.00	100.00
*p* values from two-way ANOVA						
		Prot		Prob	Prot × Prob	
IWB		0.301		0.834	0.560	
FBW		<0.001		<0.001	<0.001	
AWG		<0.001		<0.001	<0.001	
WG%		<0.001		<0.001	<0.001	
SGR		<0.001		<0.001	<0.001	
FCR		<0.001		<0.001	<0.001	
FI		0.031		0.372	0.429	
SR		-		-	-	

Note: The values are shown as the means ± SDs of three replicates (*n* = 3). Different lowercase superscript letters within a row at each probiotic level indicate a statistically significant difference, whereas different uppercase superscript letters within a column at each protease level indicate a statistically significant difference in response to probiotic levels. Abbreviations: IWB = Initial body weight; FBW = Final body weight; AWG = Average weight gain; WG% = weight gain%; SGR = Specific growth rate; FCR = feed conversion ratio; FI = Feed intake; SR = Survival rate.

**Table 3:** Interactive effects of dietary protease at different probiotic concentrations on the proximate analysis of striped catfish.

Parameters (%)	Protease (g/kg)	Probiotics (g/kg) Levels				
		0.0	1.0	2.0	3.0	4.0
Moisture	0.5	75.71 ± 0.11	75.59 ± 0.19	75.32 ± 0.70	75.36 ± 0.44	75.82 ± 0.10
	1.0	75.52 ± 0.50	75.58 ± 0.47	75.53 ± 1.06	74.97 ± 0.19	75.62 ± 0.57
Crude Protein	0.5	16.89 ± 0.96	16.96 ± 0.54	17.24 ± 0.24	17.14 ± 0.20	17.26 ± 0.42
	1.0	17.42 ± 0.41	17.16 ± 0.44	17.75 ± 0.91	17.59 ± 0.54	17.58 ± 0.60
Crude Fat	0.5	3.70 ± 0.10	3.75 ± 0.33	3.93 ± 0.61	3.66 ± 0.58	3.73 ± 0.38
	1.0	3.72 ± 0.11	3.90 ± 0.16	3.92 ± 0.18	4.02 ± 0.19	4.11 ± 0.34
Ash content	0.5	2.65 ± 0.90	2.78 ± 0.53	2.92 ± 0.61	2.88 ± 0.69	3.03 ± 0.53
	1.0	2.29 ± 0.32	3.11 ± 0.44	2.66 ± 0.43	3.23 ± 0.49	2.63 ± 0.48
*p* values from two-way ANOVA						
		Prot		Prob		Prob × Prot
Moisture		0.554		0.422		0.888
Crude Protein		0.073		0.667		0.986
Crude Fat		0.175		0.820		0.777
Ash Content		0.739		0.488		0.634

Note: The values are shown as the means ± SDs of three replicates (*n* = 3). Values without superscript letters indicate no statistically significant differences.

**Table 4:** Interactive effects of dietary protease at different probiotic concentrations on digestive enzyme activities in striped catfish.

Parameters (U/mg Protein)	Protease (g/kg)	Probiotics (g/kg) Levels					
		0.0	1.0	2.0	3.0		4.0
Amylase	0.5	1.19 ± 0.05 ^Bc^	1.41 ± 0.04 ^Bb^	1.69 ± 0.07 ^Aa^	1.77 ± 0.04 ^Aa^		1.83 ± 0.11 ^Aa^
	1.0	1.35 ± 0.01 ^Ac^	1.53 ± 0.01 ^Aa^	1.51 ± 0.01 ^Ba^	1.42 ± 0.01 ^Bb^		1.27 ± 0.01 ^Bd^
Protease	0.5	8.09 ± 0.06 ^Bc^	8.43 ± 0.05 ^Bb^	8.72 ± 0.08 ^Aa^	8.80 ± 0.04 ^Aa^		8.84 ± 0.11 ^Aa^
	1.0	8.25 ± 0.01 ^Ad^	8.56 ± 0.03 ^Aa^	8.54 ± 0.03 ^Ba^	8.42 ± 0.01 ^Bb^		8.34 ± 0.02 ^Bc^
Lipase	0.5	2.34 ± 0.02 ^Bc^	2.51 ± 0.05 ^Bb^	2.62 ± 0.03 ^a^	2.69 ± 0.03 ^Aa^		2.72 ± 0.03 ^Aa^
	1.0	2.44 ± 0.03 ^Ac^	2.68 ± 0.01 ^Aa^	2.64 ± 0.00 ^a^	2.57 ± 0.01 ^Bb^		2.35 ± 0.03 ^Bd^
*p* values from two-way ANOVA							
		Prot		Prob		Prot × Prob	
Amylase		<0.001		<0.001		<0.001	
Protease		<0.001		<0.001		<0.001	
Lipase		0.003		<0.001		<0.001	

Note: The values are shown as the means ± SDs of three replicates (*n* = 3). Different lowercase superscript letters within a row at each probiotic level indicate a statistically significant difference, whereas different uppercase superscript letters within a column at each protease level indicate a statistically significant difference in response to probiotic levels.

**Table 5:** Interactive effects of dietary protease at different probiotic concentrations on antioxidant enzyme activities in striped catfish.

Parameters	Protease (g/kg)	Probiotics (g/kg) Levels				
		0.0	1.0	2.0	3.0	4.0
SOD (U/mg protein)	0.5	4.24 ± 0.05 ^Ac^	4.49 ± 0.07 ^Bb^	4.92 ± 0.08 ^Ba^	5.03 ± 0.10 ^Aa^	5.05 ± 0.03 ^Aa^
	1.0	4.05 ± 0.05 ^Bd^	5.23 ± 0.05 ^Aa^	5.13 ± 0.06 ^Aa^	4.39 ± 0.03 ^Bb^	4.25 ± 0.05 ^Bc^
CAT (U/mg protein)	0.5	70.47 ± 0.06 ^Bc^	74.52 ± 0.08 ^Bb^	79.38 ± 0.03 ^Ba^	79.39 ± 0.03 ^Aa^	79.42 ± 0.03 ^Aa^
	1.0	77.15 ± 0.03 ^Ac^	79.49 ± 0.05 ^Aa^	79.41 ± 0.05 ^Aa^	78.08 ± 0.04 ^Bb^	76.25 ± 0.03 ^Bd^
GPx (μU/mg prot)	0.5	91.17 ± 0.05 ^Bc^	92.29 ± 0.04 ^Bb^	93.35 ± 0.01 ^Ba^	93.41 ± 0.01 ^Aa^	93.55 ± 0.01 ^Aa^
	1.0	93.22 ± 0.11 ^Ab^	94.34 ± 0.05 ^Aa^	94.24 ± 0.04 ^Aa^	92.22 ± 0.06 ^Bc^	91.37 ± 0.04 ^Bd^
MDA (mg/g prot)	0.5	3.56 ± 0.05 ^Aa^	3.13 ± 0.04 ^Ab^	2.56 ± 0.03 ^Ac^	2.55 ± 0.03 ^Bc^	2.53 ± 0.05 ^Bc^
	1.0	3.05 ± 0.03 ^Bc^	2.42 ± 0.03 ^Bd^	2.45 ± 0.06 ^Bd^	3.24 ± 0.02 ^Ab^	3.35 ± 0.03 ^Aa^
*p* values from two-way ANOVA						
		Prot		Prob		Prot × Prob
SOD		<0.001		<0.001		<0.001
CAT		<0.001		<0.001		<0.001
GPx		<0.001		<0.001		<0.001
MDA		0.024		<0.001		<0.001

Note: The values are shown as the means ± SDs of three replicates (*n* = 3). Different lowercase superscript letters within a row at each probiotic level indicate a statistically significant difference, whereas different uppercase superscript letters within a column at each protease level indicate a statistically significant difference in response to probiotic levels. Abbreviations: CAT = catalase; MDA = malondialdehyde; SOD = superoxide dismutase; GPx = glutathione peroxidase.

**Table 6:** Interactive effects of dietary protease at different probiotic concentrations on hematological parameters in striped catfish.

Parameters	Protease (g/kg)	Probiotics (g/kg) Levels					
		0.0	1.0	2.0	3.0		4.0
RBCs (10^6^ μL^−1^)	0.5	2.16 ± 0.03 ^Bc^	2.35 ± 0.02 ^Bb^	2.44 ± 0.02 ^Ba^	2.48 ± 0.04 ^Aa^		2.51 ± 0.01 ^Aa^
	1.0	2.34 ± 0.05 ^Ab^	2.55 ± 0.03 ^Aa^	2.53 ± 0.05 ^Aa^	2.22 ± 0.03 ^Bc^		2.05 ± 0.03 ^Bd^
WBCs (10^3^ μL^−1^)	0.5	5.87 ± 0.05 ^a^	5.71 ± 0.02 ^Ab^	5.42 ± 0.01 ^c^	5.48 ± 0.08 ^c^		5.55 ± 0.03 ^Ac^
	1.0	5.84 ± 0.03 ^b^	5.34 ± 0.02 ^Bd^	5.39 ± 0.03 ^d^	5.51 ± 0.05 ^c^		5.95 ± 0.03 ^Ba^
HB (g/dL)	0.5	8.15 ± 0.02 ^Bc^	8.36 ± 0.04 ^Bb^	8.45 ± 0.02 ^Ba^	8.49 ± 0.04 ^Aa^		8.52 ± 0.02 ^Aa^
	1.0	8.38 ± 0.03 ^Ab^	8.57 ± 0.04 ^Aa^	8.54 ± 0.05 ^Aa^	8.22 ± 0.04 ^Bc^		8.05 ± 0.03 ^Bd^
HCT (%)	0.5	22.06 ± 0.02 ^Bb^	22.18 ± 0.01 ^Bc^	22.31 ± 0.05 ^Ba^	22.38 ± 0.03 ^a^		22.40 ± 0.02 ^Aa^
	1.0	22.19 ± 0.02 ^Ac^	22.46 ± 0.02 ^Aa^	22.47 ± 0.00 ^Aa^	22.35 ± 0.03 ^b^		22.07 ± 0.05 ^Bd^
*p* values from two-way ANOVA							
		Prot		Prob		Prot × Prob	
RBCs		0.002		<0.001		<0.001	
WBCs		0.902		<0.001		<0.001	
HB		0.008		<0.001		<0.001	
HCT		0.001		<0.001		<0.001	

Note: The values are shown as the means ± SDs of three replicates (*n* = 3). Different lowercase superscript letters within a row at each probiotic level indicate a statistically significant difference, whereas different uppercase superscript letters within a column at each protease level indicate a statistically significant difference in response to probiotic levels. Abbreviations: HCT = hematocrit; WBCs = white blood cells; RBCs = red blood cells; HB = hemoglobin.

**Table 7:** Interactive effects of dietary protease at different probiotic concentrations on the serum biochemical and immunological parameters of striped catfish.

Parameters	Protease (g/kg)	Probiotics (g/kg) Levels					
		0.0	1.0	2.0	3.0		4.0
ALP (U/L)	0.5	31.56 ± 0.34 ^Aa^	30.25 ± 0.20 ^Ab^	28.09 ± 0.22 ^Bc^	28.29 ± 0.13 ^Bc^		28.47 ± 0.03 ^Bc^
	1.0	29.07 ± 0.04 ^Bd^	28.27 ± 0.05 ^Bc^	28.37 ± 0.05 ^Ac^	30.15 ± 0.03 ^Ab^		31.05 ± 0.03 ^Aa^
ALT (U/L)	0.5	84.71 ± 0.60 ^Aa^	82.04 ± 0.03 ^Ab^	80.03 ± 0.02 ^Ac^	80.05 ± 0.02 ^Bc^		80.06 ± 0.02 ^Bc^
	1.0	83.05 ± 0.04 ^Bd^	79.05 ± 0.03 ^Bc^	79.13 ± 0.06 ^Bc^	80.91 ± 0.11 ^Ab^		84.59 ± 0.58 ^Aa^
AST (U/L)	0.5	15.83 ± 0.02 ^Aa^	15.64 ± 0.05 ^Ab^	14.45 ± 0.06 ^Bc^	14.46 ± 0.11 ^Bc^		14.50 ± 0.22 ^Bc^
	1.0	15.20 ± 0.07 ^Bb^	14.54 ± 0.08 ^Bd^	14.64 ± 0.07 ^Ad^	15.05 ± 0.04 ^Ac^		15.35 ± 0.05 ^Aa^
Lyz (U/ml)	0.5	56.74 ± 0.51 ^Bc^	62.88 ± 0.50 ^Bb^	65.41 ± 0.08 ^Aa^	65.27 ± 0.05 ^Aa^		65.19 ± 0.03 ^Aa^
	1.0	59.13 ± 0.04 ^Ad^	64.38 ± 0.06 ^Aa^	64.28 ± 0.04 ^Ba^	63.18 ± 0.03 ^Bb^		62.27 ± 0.05 ^Bc^
IgM (mg/ml)	0.5	3.18 ± 0.04 ^Bc^	4.09 ± 0.06 ^Bb^	5.81 ± 0.00 ^Aa^	5.53 ± 0.25 ^Aa^		5.49 ± 0.29 ^Aa^
	1.0	3.41 ± 0.03 ^Ac^	5.59 ± 0.07 ^Aa^	5.35 ± 0.29 ^Ba^	4.64 ± 0.05 ^Bb^		4.17 ± 0.05 ^Bd^
*p* values from two-way ANOVA							
		Prot		Prob		Prot × Prob	
ALP		0.322		<0.001		<0.001	
ALT		0.739		<0.001		<0.001	
AST		0.260		<0.001		<0.001	
Lyz		<0.001		<0.001		<0.001	
IgM		0.004		<0.001		<0.001	

Note: The values are shown as the means ± SDs of three replicates (*n* = 3). Different lowercase superscript letters within a row at each probiotic level indicate a statistically significant difference, whereas different uppercase superscript letters within a column at each protease level indicate a statistically significant difference in response to probiotic levels. Abbreviations: ALP = alkaline phosphatase; ALT = alanine aminotransferase; AST = aspartate aminotransferase; IgM = immunoglobulin M; Lyz = lysozyme.

## Data Availability

The data supporting the findings of this study are available from the corresponding authors upon reasonable request.
